# Learning Curve Analysis of Laparoscopic Intracorporeal Sac Transection and Purse-String Suture for Pediatric Inguinal Hernia Repair

**DOI:** 10.3390/children13050628

**Published:** 2026-04-30

**Authors:** Hee-Beom Yang

**Affiliations:** 1 Department of Surgery, Seoul National University Bundang Hospital, 82 Gumi-ro 173beon-gil, Bundang-gu, Seongnam 13620, Republic of Korea; eeulere@snubh.org; Tel.: +82-31-787-7118; Fax: +82-50-4267-6417; 2 Department of Surgery, Seoul National University College of Medicine, Seoul 03080, Republic of Korea

**Keywords:** inguinal hernia, pediatrics, CUSUM analysis, learning curve

## Abstract

**Highlights:**

**What are the main findings?**

- Cumulative sum (CUSUM) analysis determined that technical proficiency for laparoscopic intracorporeal sac transection with purse-string suture (LIST-PS) is achieved after 233 cases.
- Mean operative time decreased significantly from 70.6 min in the learning phase to 52.0 min in the proficiency phase, while the recurrence rate remained stable at 1.7% across both periods.

**What are the implications of the main finding?**

- The study demonstrates that high surgical safety and low recurrence rates can be maintained throughout the learning process, even before maximum operative efficiency is reached.
- Technical improvement follows a parallel trajectory across all patient groups, confirming that LIST-PS is a reliable and definitive approach for both standard and high-risk pediatric patients, such as preterm infants.

**Abstract:**

**Background**: Although various laparoscopic techniques are available for pediatric hernia repair, the learning curve for laparoscopic intracorporeal sac transection with purse-string suture (LIST-PS) has not yet been established. Considering that intracorporeal suturing is technically more challenging than extracorporeal knotting, an objective assessment of surgical competence is crucial. The study aimed to evaluate the learning curve and safety profile of LIST-PS performed by a single surgeon in a large series of pediatric patients. **Methods**: A retrospective analysis of 469 pediatric patients treated between March 2019 and December 2025 was conducted. The learning curve was assessed using the cumulative sum (CUSUM) analysis of operative times. The cohort was divided into phases 1 (learning) and 2 (proficiency) based on the CUSUM peak. Additionally, a high-risk subgroup (preterm infants aged < 1 year) was compared with a control group to evaluate the impact of patient complexity on surgical progress. **Results**: The mean patient age was 3.4 ± 3.5 years, and 29.4% were born prematurely. The CUSUM analysis identified a peak at case 233, marking the transition to proficiency. The mean operative time decreased significantly from 70.6 ± 26.3 min in phase 1 to 52.0 ± 16.5 min in phase 2 (*p* < 0.001). Despite the reduction in operative time, the recurrence rate remained stable at 1.7% in both phases (*p* > 0.999). In a subgroup analysis, the high-risk group (preterm infants and infants < 1 year) required longer operative times (73.5 ± 32.8 min vs. 57.7 ± 19.2 min; *p* < 0.001) but showed no significant difference in recurrence compared to the control group (0.96% vs. 1.92%; *p* = 0.691). **Conclusions**: Technical proficiency in LIST-PS was achieved after 233 cases. Although high-risk patients consistently required more time, the surgeon’s improvement followed a parallel trajectory across all risk levels, maintaining high surgical safety throughout the learning process.

## 1. Introduction

Pediatric inguinal hernia is one of the most common surgical conditions encountered in children, primarily due to the failed closure of the processus vaginalis [1]. The estimated prevalence ranges significantly, occurring in approximately 0.8% to 4.4% of full-term infants, with a markedly higher incidence observed in premature infants, reaching up to 30% [2]. If left untreated, it poses a substantial risk of incarceration and subsequent bowel or gonadal ischemia, making surgical intervention the standard of care [3,4].

Historically, the “gold standard” for repair has been open high ligation of the hernia sac. However, the last few decades have seen a significant evolution in surgical techniques with the advent and refinement of minimally invasive surgery [5,6]. Laparoscopic inguinal hernia repair has gained widespread popularity due to its distinct advantages, including excellent visualization of the internal ring, the ability to evaluate and treat a contralateral patent processus vaginalis simultaneously, and improved cosmetic outcomes [6].

Various techniques have emerged in the field of laparoscopic approaches, ranging from percutaneous internal ring suturing to more complex intracorporeal suturing. Among these, the intracorporeal suture and knot-tying technique is particularly noteworthy because it most closely mimics the principles of traditional open high ligation [7]. By performing the ligation from within the peritoneal cavity, surgeons can achieve precise closure of the internal ring while minimizing trauma to the surrounding cord structures [8].

Despite its theoretical benefits and increased adoption, the intracorporeal technique is widely recognized as technically demanding and requires advanced laparoscopic skills [9]. Surprisingly, there is a lack of comprehensive data on the learning curve associated with this procedure in children. Understanding the transition from the initial learning phase to surgical proficiency is crucial for optimizing operative efficiency and ensuring patient safety [10].

This study aimed to evaluate the learning curve of a single surgeon performing laparoscopic intracorporeal sac dissection and purse-string sutures for hernia repair. Using cumulative sum (CUSUM) analysis, the study sought to identify the point of technical proficiency and compare the surgical outcomes between the early learning and subsequent proficiency phases. Additionally, the study investigated the impact of the learning process on high-risk groups, such as preterm infants and those under one year of age, to determine whether technical improvement remains consistent across varying levels of patient complexity.

## 2. Materials and Methods

We followed the STROBE checklist for observational studies in the design and reporting of this study.

### 2.1. Study Design and Population

**Figure 1:**
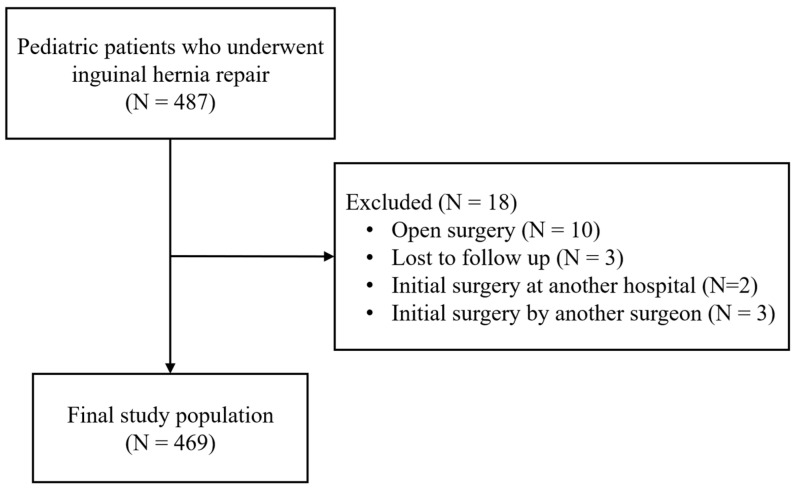
Flowchart of patient selection and study enrollment.

This retrospective cohort study aimed to evaluate the surgical learning curve and clinical outcomes of pediatric inguinal hernia repair performed by a single surgeon. The study protocol was reviewed and approved by the Institutional Review Board (Approval Number: B-2602-1027-104), and the requirement for informed consent was waived because of the retrospective nature of the study. All pediatric patients who underwent hernia repair between 1 March 2019 and 31 December 2025 were identified by a search of the electronic medical record system. A consecutive series of patients was included in this study to avoid potential selection bias. Of the initial 487 pediatric patients who underwent inguinal hernia repair, 18 were excluded based on the following criteria: loss to follow-up (N = 3), open surgical approach (N = 10), and initial surgery performed by other providers (N = 5). A total of 469 patients were included in the final analysis (Figure 1).

The collected data included demographic information such as age at surgery, weight, and sex. Clinical variables included prematurity (gestational age < 37 weeks), neonatal intensive care unit (NICU) admission status at the time of surgery, and preoperative laterality. The surgical variables included the type of admission (daycare vs. inpatient), urgency of the procedure (elective vs. emergency), postoperative laterality, and total operative time, which was defined as the time from the initial skin incision to the completion of skin closure. Daycare surgery was primarily indicated for otherwise healthy infants who had reached a postmenstrual age of at least 60 weeks at the time of surgery. All patients underwent routine follow-up at 1 week and 3 months postoperatively. For patients who underwent surgery at less than 3 months of age, an additional follow-up was performed at 9 months. To assess the influence of patient complexity on surgical proficiency and outcomes, the patients were categorized into two groups. The high-risk group included patients who were born prematurely (<37 weeks) and were aged < 1 year at the time of surgery. The control group consisted of all pediatric patients who did not meet either criterion.

### 2.2. Surgical Procedure

**Figure 2:**
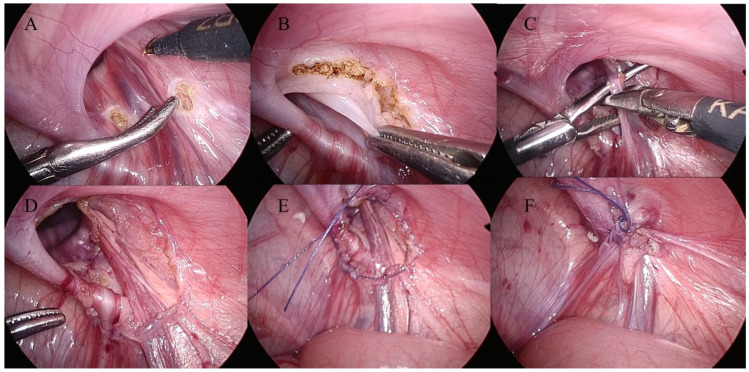
Key surgical steps of the laparoscopic intracorporeal sac transection and purse-string suture: (**A**) Identification of the internal inguinal ring and initiation of the sac dissection. (**B**) Dissection along the superior margin of the internal ring. (**C**) Careful dissection of the inferior margin adjacent to the vas deferens and gonadal vessels. (**D**) Completion of the hernia sac dissection. (**E**) High ligation using a figure-of-eight purse-string suture. (**F**) Final appearance after complete closure of the internal ring.

Although the surgeon was proficient in general laparoscopic maneuvers, the specific sequence of 360-degree sac dissection and intracorporeal knot tying was initiated exclusively in case 1 in this series. All procedures were performed with patients in the supine position under general anesthesia. The surgeon was positioned at the head of the patient, facing the caudal direction to facilitate an optimal ergonomic view and instrument manipulation. Pneumoperitoneum was established and maintained at a pressure of 10–12 mmHg via CO_2_ insufflation, with a flow rate set at 3 L/min. For laparoscopic access, a 5-mm trocar was inserted through the umbilicus, and two 3 mm trocars were placed bilaterally in the lower quadrants. In patients with a concomitant umbilical hernia, the hernia sac was excised first, and the resulting fascial opening was used for the insertion of a 5 mm trocar. For the suture material, 4-0 Polysorb (Medtronic, Minneapolis, MN, USA) or 5-0 Vicryl (Ethicon, Somerville, NJ, USA) sutures were used and cut to a length of 10–12 cm. This suture was introduced into the abdominal cavity via a direct puncture through the abdominal skin using a needle. For preoperatively diagnosed hernias, the hernial sac was meticulously dissected from the cord structures; however, excision of the sac was not performed. The internal inguinal ring was closed using an intracorporeal figure-of-eight purse-string suture, with meticulous care taken to avoid injury to the vas deferens and gonadal vessels in male patients. In female patients, the round ligament was divided and fixed to the internal ring during closure. In cases of incidental contralateral patent processus vaginalis or patent canal of Nuck, an intracorporeal figure-of-eight purse-string suture was performed directly without dissection, regardless of the opening size. In patients with non-communicating hydroceles, the fluid was aspirated externally using an 18-gauge needle. The knot was tied intracorporeally using an instrument-tying technique consisting of an initial double-throw knot followed by two additional single-throw knots. Prior to tightening the knot, the assistant applied manual pressure on the groin area to completely evacuate any trapped air from the hernia tract. After the tie was secured, the site was carefully inspected to confirm the absence of re-inflation, ensuring an airtight and definitive closure of the internal ring. The umbilical wound was closed layer-by-layer with fascial closure, followed by knot-burying subcuticular skin sutures. For the 3 mm trocar sites, closure was achieved by applying three Steri-Strips (3M, St. Paul, MN, USA) to each incision without the need for additional suturing (Figure 2).

### 2.3. Statistical Analysis and Learning Curve Evaluation

Continuous variables are presented as mean ± standard deviation (SD), and categorical variables are presented as frequencies and percentages. To compare patient characteristics and surgical outcomes between groups, Student’s *t*-test was used for continuous data, while the chi-square test or Fisher’s exact test was employed for categorical data, as appropriate. The surgical learning curve was primarily evaluated using cumulative sum (CUSUM) analysis of the operative times. Operative time was selected as the primary performance metric for CUSUM analysis because it provides a continuous and sensitive measure of technical proficiency, particularly in procedures like pediatric hernia repair, where major complication rates are too low to provide sufficient statistical power for learning curve modeling. The CUSUM for operative time (CUSUM-OT) was calculated as $CUSUM_{OT}=\sum_{i=1}^{n}{(X}_{i}-\mu$), where *X_i_* is the operative time for an individual case, and μ is the mean operative time for all cases in the series. The peak of the CUSUM curve was used to identify the specific number of cases representing the transition from the learning phase (phase 1) to the proficiency phase (phase 2). Additionally, a moving average (N = 30) was calculated to visualize the chronological trends in operative time. Linear regression analysis was performed to compare the slopes of the learning curves between the high-risk and control groups to determine whether the rate of technical improvement was consistent across different levels of patient complexity. All statistical analyses were performed using R software, version 4.3.1 (R Foundation for Statistical Computing, Vienna, Austria), and a *p*-value < 0.05 was considered statistically significant.

## 3. Results

### 3.1. Baseline Characteristics and Overall Surgical Outcomes

**Figure 3:**
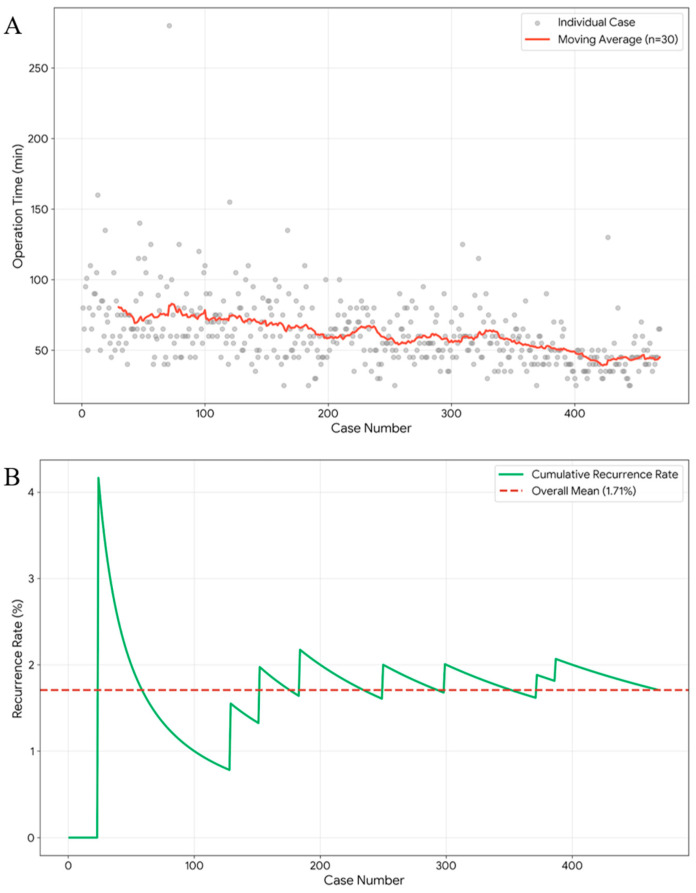
Sequential operative time and recurrence cases: (**A**) Chronological operative times for 469 patients with a moving average trend line. (**B**) Cumulative recurrence rate.

The study included 469 patients who met the inclusion criteria. The cohort had a mean age of 3.4 ± 3.5 years and a mean weight of 15.0 ± 11.5 kg. Male patients comprised most of the population (N = 306, 65.2%). The clinical complexity of the study population was notable: 29.4% of patients were born preterm (<37 weeks’ gestation), and 3.6% were NICU inpatients at the time of surgery. Preoperatively, most patients presented with unilateral hernias (90.8%), with right-sided hernias being slightly more common than left-sided hernias. However, following intraoperative laparoscopic evaluation of the contralateral side, the proportion of bilateral repairs significantly increased to 48.6%. Regarding surgical settings, 66.5% of procedures were performed as daycare surgeries, whereas emergency surgery was required for 2.6% of the patients. The overall mean operative time was 61.2 ± 23.8 min. The recurrence rate was 1.7% (N = 8) observed during a median follow-up period of 99 days (Table 1) (Figure 3).

### 3.2. CUSUM Analysis and Learning Curve Phases

**Figure 4:**
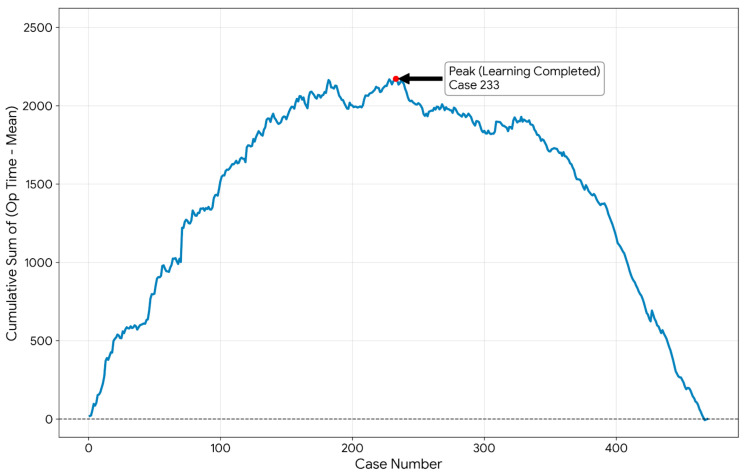
CUSUM analysis for operative time. The cumulative sum (CUSUM) curve represents the cumulative difference between individual and mean operative times. The peak at case 233 indicates the cutoff point between the learning (phase 1) and the proficiency (phase 2) phases.

To characterize the surgical learning curve, a CUSUM analysis was performed based on the operative times. The CUSUM-OT curve showed a distinct peak at case 233 (Figure 4). Based on the peak of the CUSUM-OT curve, the cases were divided into two phases: the learning (phase 1, cases 1–233) and proficiency (phase 2, cases 234–469) phases. A comparative analysis between the learning and proficiency phases revealed no statistically significant differences in patient demographics, including age (*p* = 0.868), weight (*p* = 0.782), or sex (*p* = 0.628). Similarly, the proportions of premature births, NICU admissions, postoperative laterality, and emergency cases remained consistent across both phases (all *p* > 0.05). The primary difference between the phases was the significant reduction in operative time. The mean operative time decreased significantly from 70.6 ± 26.3 min in phase 1 to 52.0 ± 16.5 min in phase 2 (*p* < 0.001). Despite the increased speed in the later phase, clinical safety was maintained, and the recurrence rate was identical between phases 1 and 2 at 1.7% (N = 4 for each, *p* > 0.999) (Table 2).

### 3.3. Analysis of the High-Risk Subgroup

**Figure 5:**
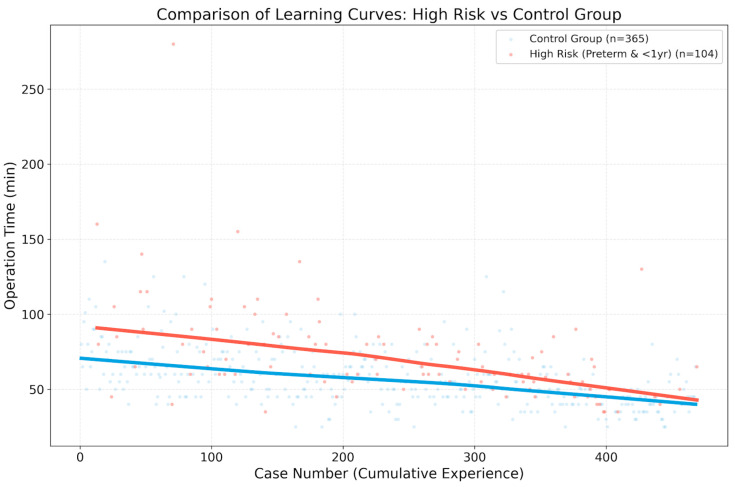
Comparison of operative time between the high-risk and control groups. The trend lines show parallel downward trajectories for both groups. This indicates consistent technical improvement regardless of patient complexity, although the high-risk group (preterm infants and infants aged < 1 year) consistently required longer operative times.

The impact of patient complexity on surgical outcomes was evaluated by comparing a high-risk group (preterm infants and infants < 1 year of age, N = 104) with a control group (N = 365). The high-risk group required significantly longer operative times compared to the control group (73.5 ± 32.8 min vs. 57.7 ± 19.2 min; *p* < 0.001). However, this increased technical difficulty did not translate into higher complication rates, as the recurrence rate showed no significant difference between the two groups (0.96% in high-risk vs. 1.92% in control; *p* = 0.691) (Table 3). Trend lines for both groups showed parallel downward trajectories (Figure 5).

## 4. Discussion

Surgical management of pediatric inguinal hernia has undergone a significant paradigm shift over the last two decades. Although open high ligation remains the classic approach, laparoscopic repair has become increasingly prevalent because of its ability to identify the contralateral patent processus vaginalis and provide superior cosmetic results. Among the various laparoscopic techniques, intracorporeal suturing with knot-tying is regarded as a very technically demanding method because it requires advanced needle handling in the restricted pelvic space of an infant or child [8]. This study aimed to define the learning curve for this specific technique using CUSUM analysis in a large cohort of 469 patients. Our findings indicate that technical proficiency is achieved after 233 cases, at which point the operative efficiency significantly improves without compromising surgical safety.

### 4.1. The Learning Curve and CUSUM Analysis

The learning curve in surgery describes the relationship between the number of procedures performed and the resulting improvement in performance, which is often measured by operative time or complication rates [10,11,12,13,14]. Traditional methods for assessing learning curves often rely on arbitrary divisions of the study period, which can mask the true point of transition. In contrast, the CUSUM analysis used in this study provides a mathematically rigorous method to identify the peak of the learning process. The analysis identified a peak after 233 cases, dividing the experience into learning (phase 1) and proficiency (phase 2) phases. This number is notably higher than that reported for other laparoscopic techniques. For instance, studies on the percutaneous internal ring suture (PIRS) technique often suggest a shorter learning curve of approximately 30–50 cases [13,15,16]. This discrepancy is expected, as PIRS is a simplified extracorporeal method. In contrast, intracorporeal sac dissection with suturing requires mastering two-handed needle manipulation and knot-tying within a small peritoneal cavity. In addition, achieving a complete 360-degree dissection of the internal ring presents a significant technical challenge, as it requires meticulous effort to safeguard the vital cord structures situated along the inferior aspect of the ring [17,18]. Our results align closely with those of laparoscopic percutaneous extraperitoneal closure, where 125 cases are necessary to reach a plateau in operative time [14].

### 4.2. Operative Efficiency and Clinical Safety

The most striking difference between the two phases in the study was the operative time. The mean operative time decreased from 70.6 ± 26.3 min in the learning phase to 52.0 ± 16.5 min in the proficiency phase (*p* < 0.001). This significant reduction reflects an improvement in technical efficiency, better anticipation of steps, and more efficient handling of the hernial sac and suture material. It is noteworthy that patient characteristics, including age, weight, and hernia laterality, were statistically comparable between the two phases (*p* > 0.05). This indicates that the observed reduction in operative time resulted from the surgeon’s increasing proficiency rather than the selection of less complex cases. Regarding safety, the recurrence rate in the study was 1.7% overall (8 out of 469 cases). Remarkably, the recurrence rates were identical between phases 1 and 2 (1.7% vs. 1.7%, *p* > 0.999). This suggests that the quality of repair, which ensures secure high ligation, can be maintained even during the early stages of the learning curve, albeit at the cost of longer operative times. This finding is consistent with the literature, suggesting that while operative speed is a function of experience, surgical accuracy can be achieved early through rigorous adherence to technical principles [19,20]. Laparoscopic repair can be associated with rare but potentially life-threatening complications. These include bowel strangulation secondary to adhesions, port-site hernias, omental evisceration, and iliac vein puncture resulting in a retroperitoneal hematoma—some of which are unique to minimally invasive surgery [21].

### 4.3. Impact of Patient Complexity

A unique aspect of this study was the subanalysis of high-risk patients, defined as those who were premature (<37 weeks) or aged < 1 year at the time of surgery. Surgery in these infants is notoriously difficult because of the fragile nature of their tissues, extremely limited working space, and increased risk of anesthetic complications [22]. Our data showed that the high-risk group required significantly longer operative times compared to the control group (73.5 ± 32.8 min vs. 57.7 ± 19.2 min, *p* < 0.001). However, the learning curve trajectories for both groups were nearly parallel. This indicates that the technical improvements gained through experience are applicable to all patient types. Even as the surgeon becomes more proficient, high-risk cases will inherently take longer due to anatomical challenges, but the “rate” of improvement is consistent with simpler cases. This provides reassurance that a surgeon’s growth in proficiency benefits even the most vulnerable patient populations [23].

### 4.4. Comparisons with Previous Literatures

The reported recurrence rates for laparoscopic pediatric hernia repair vary widely in the literature, ranging from 0.5% to 4% [8,24]. Our rate of 1.7% falls well within this established range. Although the mean operative time in the current study was longer than that reported in a previous study using the same procedure (61.2 min vs. 27.5 min) [25], several critical factors must be considered. First, while the current study encompassed the surgeon’s entire clinical experience, starting from case 1, the baseline proficiency level of the surgeon in the comparative study remains unclear. Second, the current cohort was characterized by significantly higher patient complexity. Specifically, the proportion of infants under 3 months of age was more than double that of the previous study (24.3% vs. 11.9%), and the rate of prematurity was markedly higher in the current series (29.4% vs. 4.3%). These substantial disparities in patient difficulty and the inclusion of a larger number of high-risk patients likely contributed to the relatively longer operative times and the more extensive learning curve observed in this study. Consequently, the findings of the current study offer a pragmatic representation of the learning curve in a clinical setting characterized by a high volume of complex, high-risk pediatric patients. This study contributes to the literature by quantifying the temporal costs of learning processes. Although a threshold of 233 cases may be perceived as extensive, the fact that the complication and recurrence rates remained low and stable during phase 1 validates the safety of adopting this technique in pediatric surgical practice.

## 5. Limitations

Despite the large sample size, this study had several limitations that warrant consideration. First, as this study reflects the experience of a single surgeon, the identified threshold of 233 cases may not be directly generalizable to other practitioners with different training backgrounds or institutional settings. Second, the retrospective nature of this study carries the inherent risk of loss to follow-up. Patients residing in distant geographical areas may have sought care at other medical institutions if a recurrence occurred, potentially leading to an underestimation of the recurrence rate. Additionally, although pediatric inguinal hernia recurrence typically manifests early, the median follow-up period of 99 days may have been insufficient to fully evaluate the long-term durability of this intracorporeal technique and capture late-onset recurrence. Finally, the absence of a direct comparison group, such as patients undergoing open repair or other laparoscopic techniques like PIRS, limits our ability to evaluate the comparative clinical outcomes and technical characteristics of the intracorporeal approach.

## 6. Conclusions

While the procedure involving laparoscopic intracorporeal sac transection and purse-string repair requires a learning curve of approximately 233 cases, it ensures a consistently low recurrence rate regardless of experience. Although initially more time-consuming, achieving proficiency significantly improves operative efficiency without compromising safety. Its reliability, even in high-risk patients, underscores its clinical utility as a safe and definitive method for pediatric hernia repair.

## Figures and Tables

**Table 1:** Patient characteristics and surgical outcomes.

	N = 469
Age at surgery (years, mean ± SD)	3.4 ± 3.5
Weight at surgery (kg, mean ± SD)	15.0 ± 11.5
Male	306 (65.2%)
Prematurity (<37 weeks)	138 (29.4%)
NICU at surgery	17 (3.6%)
Emergency surgery	12 (2.6%)
Daycare surgery	312 (66.5%)
Preoperative laterality	
Right	221 (47.1%)
Left	205 (43.7%)
Bilateral	43 (9.2%)
Postoperative laterality	
Right	141 (30.1%)
Left	100 (21.3%)
Bilateral	228 (48.6%)
Operative time (min, mean ± SD)	61.2 ± 23.8
Recurrence	8 (1.7%)
Follow-up duration (days, median, interquartile range)	99 (42–135)

NICU, neonatal intensive care unit.

**Table 2:** Comparison of patient characteristics and outcomes between the phases.

	Learning (N = 233)	Proficiency (N = 236)	*p*-Value
Age at surgery (years, mean ± SD)	3.4 ± 3.7	3.3 ± 3.3	0.868
Weight at surgery (kg, mean ± SD)	15.4 ± 12.5	14.6 ± 10.4	0.782
Male	155 (66.5%)	151 (64.0%)	0.628
Prematurity (<37 weeks)	65 (27.9%)	73 (30.9%)	0.48
NICU at surgery	10 (4.3%)	7 (3.0%)	0.47
Emergency surgery	7 (3.0%)	5 (2.1%)	0.574
Daycare surgery	161 (69.1%)	151 (64.0%)	0.282
Postoperative laterality			0.483
Right	75 (32.2%)	66 (28.0%)	
Left	51 (21.9%)	49 (20.8%)	
Bilateral	107 (45.9%)	121 (51.3%)	
Operative time (min, mean ± SD)	70.6 ± 26.3	52.0 ± 16.5	**<0.001**
Recurrence	4 (1.7%)	4 (1.7%)	>0.999

NICU, neonatal intensive care unit.

**Table 3:** Surgical outcomes in the high-risk group compared to the control group.

	High-Risk Group * (N = 104)	Control Group (N = 365)	*p*-Value
Recurrence, N (%)	1 (0.96%)	7 (1.92%)	0.691
Operative time (min, mean ± SD)	73.5 ± 32.8	57.7 ± 19.2	**<0.001**

* High-risk group: gestational age < 37 weeks and age at surgery < 1 year; Control group: all other patients.

## Data Availability

No new data were created.
